# Polar motion prediction using the combination of SSA and Copula-based analysis

**DOI:** 10.1186/s40623-018-0888-3

**Published:** 2018-07-11

**Authors:** Sadegh Modiri, Santiago Belda, Robert Heinkelmann, Mostafa Hoseini, José M. Ferrándiz, Harald Schuh

**Affiliations:** 1 GFZ German Research Centre for Geosciences, Potsdam, Germany; 2 Institute for Geodesy and Geoinformation Science, Technische Universität Berlin, Berlin, Germany; 3 EPS, Applied Mathematics Department, University of Alicante, Campus de San Vicente, Alicante, Spain; 4 Department of Civil and Environmental Engineering, Norwegian University of Science and Technology, Trondheim, Norway

**Keywords:** Copula, SSA, Polar motion, EOP, Prediction

## Abstract

The real-time estimation of polar motion (PM) is needed for the navigation of Earth satellite and interplanetary spacecraft. However, it is impossible to have real-time information due to the complexity of the measurement model and data processing. Various prediction methods have been developed. However, the accuracy of PM prediction is still not satisfactory even for a few days in the future. Therefore, new techniques or a combination of the existing methods need to be investigated for improving the accuracy of the predicted PM. There is a well-introduced method called Copula, and we want to combine it with singular spectrum analysis (SSA) method for PM prediction. In this study, first, we model the predominant trend of PM time series using SSA. Then, the difference between PM time series and its SSA estimation is modeled using Copula-based analysis. Multiple sets of PM predictions which range between 1 and 365 days have been performed based on an IERS 08 C04 time series to assess the capability of our hybrid model. Our results illustrate that the proposed method can efficiently predict PM. The improvement in PM prediction accuracy up to 365 days in the future is found to be around 40% on average and up to 65 and 46% in terms of success rate for the ${\hbox{PM}}_{x}$ and ${\hbox{PM}}_{y}$, respectively.
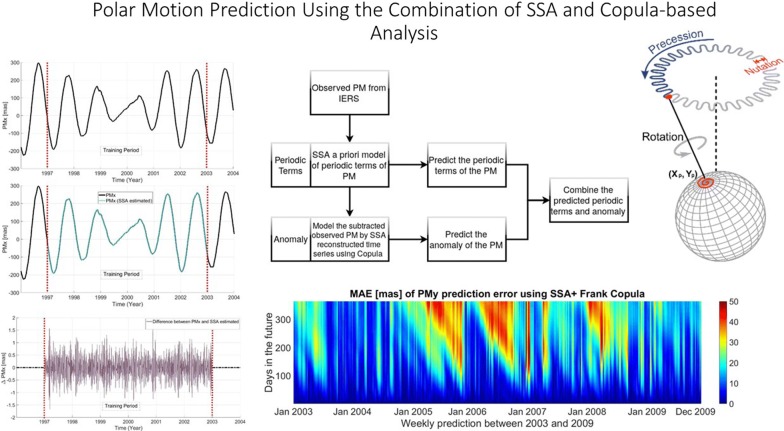

## Introduction

Polar motion (PM) describes the movement of the Earth’s rotation axis w.r.t the Earth surface. The study of PM provides valuable information for studying many geophysical and meteorological phenomena (Barnes et al. 1983; Wahr 1982, 1983; Mathews et al. 1991; Gross et al. 2003; Chen and Wilson 2005; Gross 2015; Seitz and Schuh 2010; Schuh and Böhm 2011).

Since the 1960s, highly accurate PM coordinates can be obtained by different space geodesy techniques. These techniques include: Satellite Laser Ranging (SLR) (Coulot et al. 2010), Lunar Laser Ranging (LLR) (Dickey et al. 1985), Doppler Orbitography and Radiopositioning Integrated by Satellite (DORIS) (Angermann et al. 2010), Global Navigation Satellite Systems (GNSS) (Dow et al. 2009; Byram and Hackman 2012), and very-long-baseline interferometry (VLBI) (Schuh and Schmitz-Hübsch 2000; Nilsson et al. 2010, 2011, 2014).

Accurate real-time PM is needed for high-precision satellite navigation and positioning and spacecraft tracking (Kalarus et al. 2010; Stamatakos 2017). However, the PM is not provided in real time due to the complexity of the measurement model and data processing; PM coordinates are available with a delay of hours to days (Bizouard and Gambis 2009; Schuh and Behrend 2012). Therefore, it is essential to predict the PM parameters precisely.

**Fig. 1:**
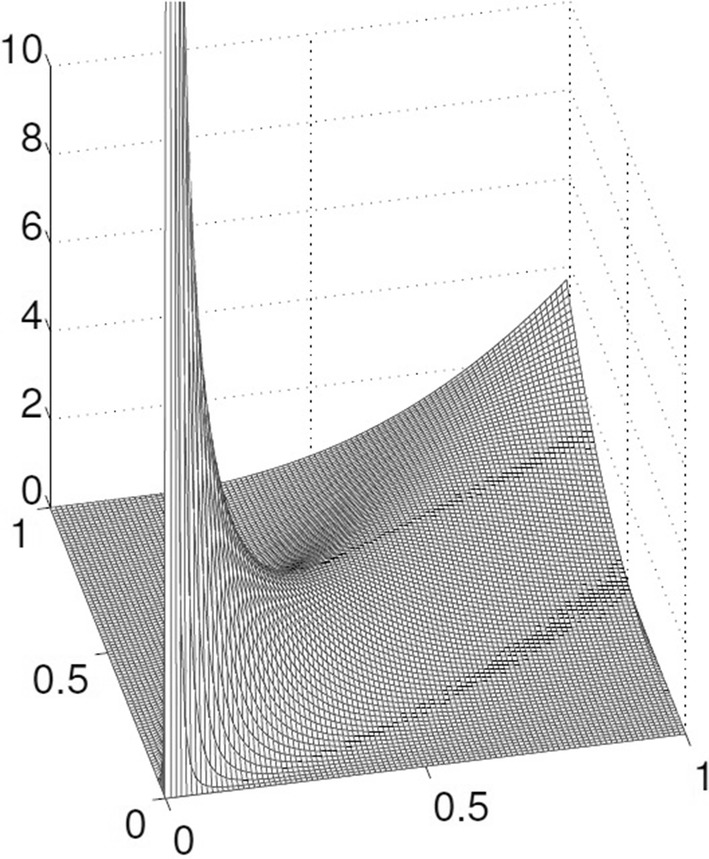
Clayton Copula with parameter $\theta = 3$. The Clayton Copula is an asymmetric Archimedean Copula; it shows greater dependence in the lower tail than in the upper tail

**Fig. 2:**
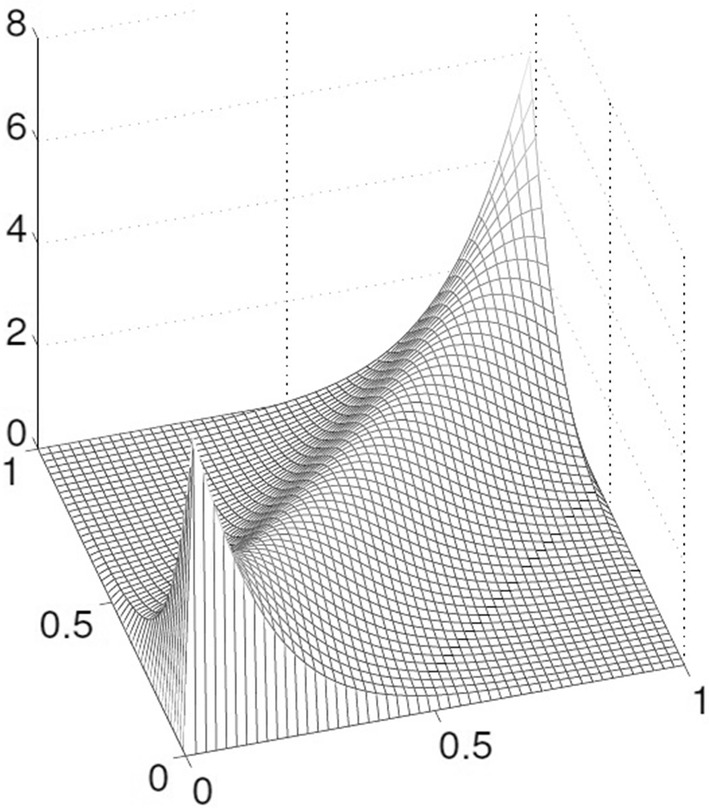
Frank Copula with parameter $\theta = 8$. The Frank Copula is a symmetric Archimedean Copula

**Fig. 3:**
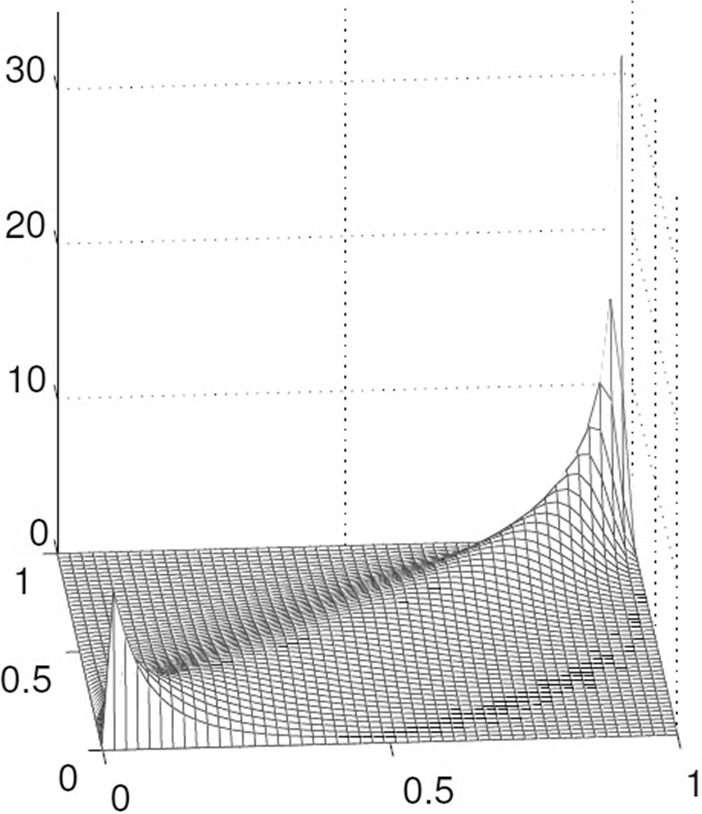
Gumbel Copula with parameter $\theta = 3$. Gumbel Copula can capture strong upper tail dependency and weak lower tail dependency

**Fig. 4:**
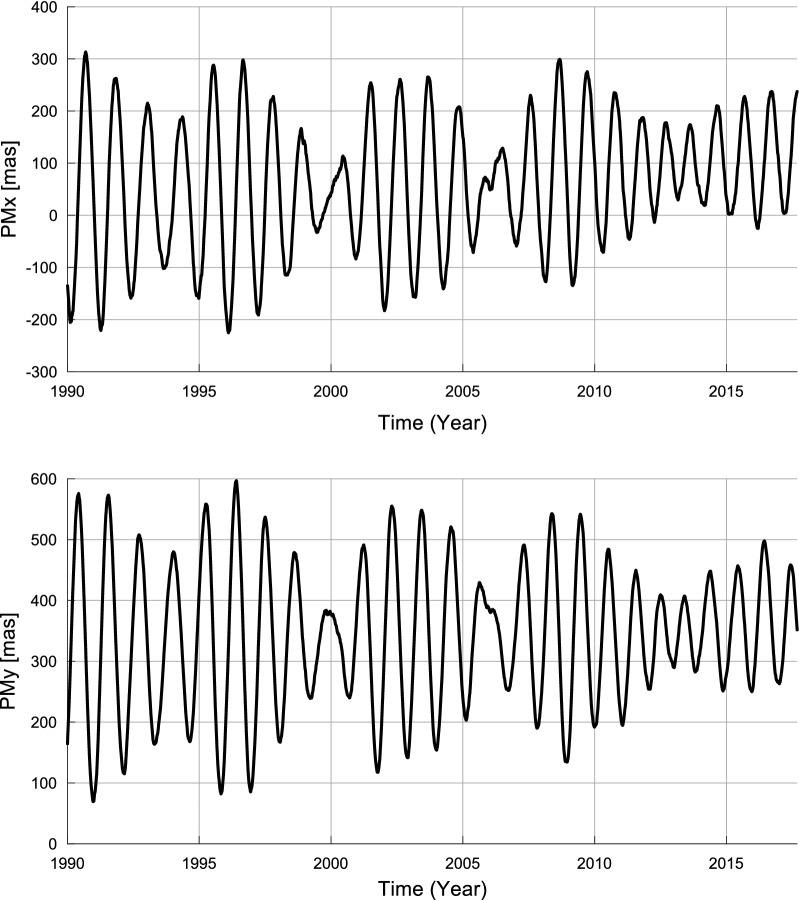
Daily PM time series from 1990 to the present

**Fig. 5:**
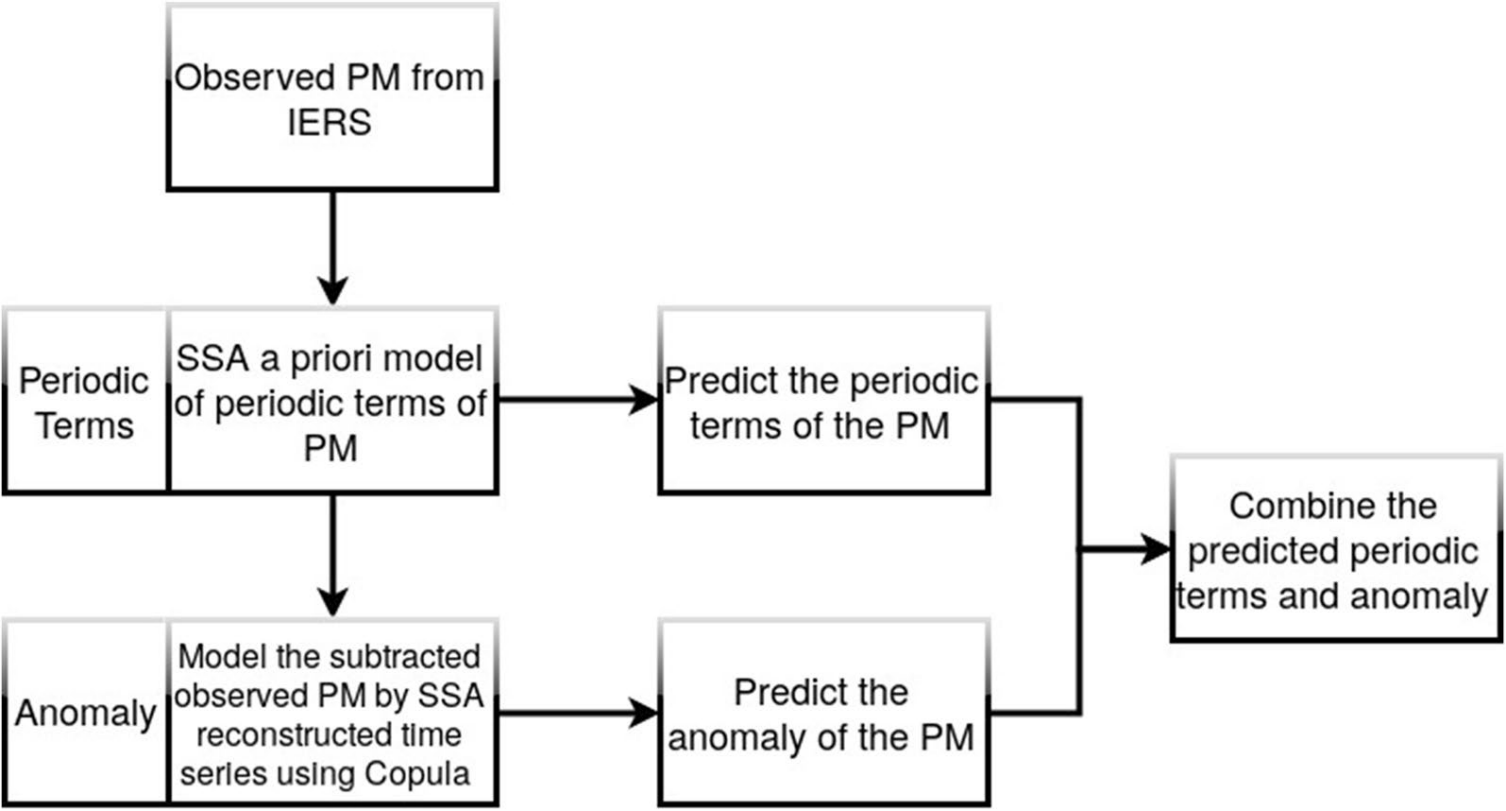
Scheme of the SSA+Copula model for PM prediction

Different methods and means have been investigated and applied for PM prediction such as least squares (LS) collocation (Włodzimierz 1990), spectral analysis and LS extrapolation method (Akulenko et al. 2002), LS extrapolation of a harmonic model and autoregressive (AR) prediction (Kosek et al. 1998, 2007; Xu et al. 2012), wavelets and fuzzy inference systems (Akyilmaz and Kutterer 2004; Akyilmaz et al. 2011) modeling and forecasting excitation functions (Chin et al. 2004), Kalman filter with atmospheric angular momentum forecasts (Freedman et al. 1994), and artificial neural network (ANN) (Schuh et al. 2002; Kalarus and Kosek 2004). The Earth orientation parameters prediction comparison campaign (EOP PCC) took place within (2005–2009), and the results demonstrate that there is no particular method superior to other for all prediction intervals (Kalarus et al. 2010). Among these methods, the combination of LS and AR process is considered to be one of the most effective for PM prediction (Kalarus et al. 2010). The mentioned combination method achieved reasonable results for short-term forecasting. However, due to the complexity of the PM excitation model, it is not able to reproduce the time variation of the periodic terms that influence the long-term predictive accuracy of PM. Consequently, a new prediction method is required that could bring us significantly closer to meeting the accuracy goals pursued by the Global Geodetic Observing System (GGOS) of the International Association of Geodesy (IAG), i.e., 1 mm accuracy and 0.1 mm/year stability on global scales in terms of the ITRF defining parameters (Plag and Pearlman 2009). Therefore new techniques or a combination of the existing methods need to be investigated for improving the efficiency of the predicted PM considering the time variation of the periodic terms and the trend. In this study, we examined the combination of singular spectrum analysis (SSA) and Copula-based analysis to predict PM. SSA is not constrained by the assumptions of using predetermined functions such as sine wave as the base; it rather exploits data-driven base functions for extracting fundamental components of the time series and applies a classification method to explore the relationship between the derived elements (Broomhead and King 1986; Vautard et al. 1992; Zotov 2005). The Copula method operates linear and nonlinear dependency between variables, and it is a potent and efficient tool for dealing with multi-dimensional data and modeling the relationship between parameters (Joe 1997). The combination of SSA and Copula-based methods will be applied for the first time as a novel stochastic tool for PM determination.

**Fig. 6:**
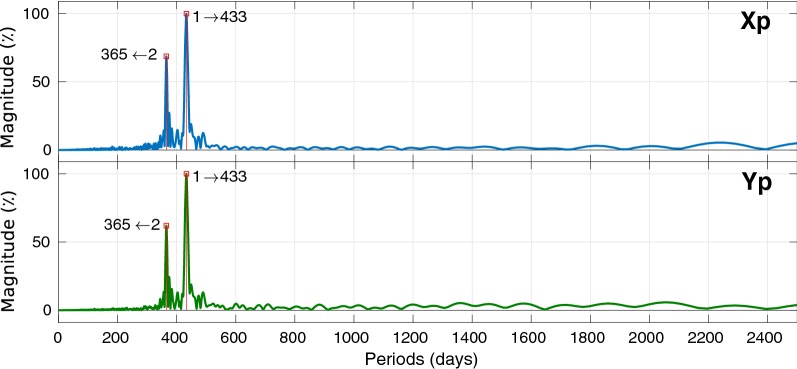
Spectral analysis of the ${\hbox{PM}}_{x}$ (up), ${\hbox{PM}}_{y}$ (down) using fast Fourier transform (FFT)

**Fig. 7:**
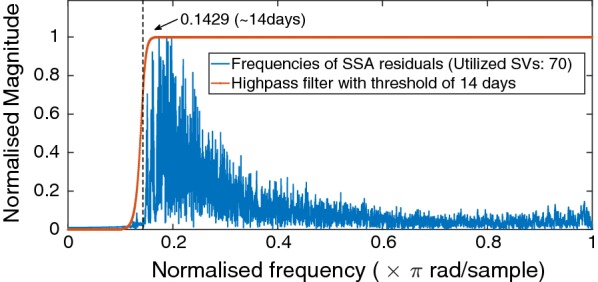
Number of singular values and vectors applied in modeling polar motion to achieve 1 mas degree of accuracy

**Fig. 8:**
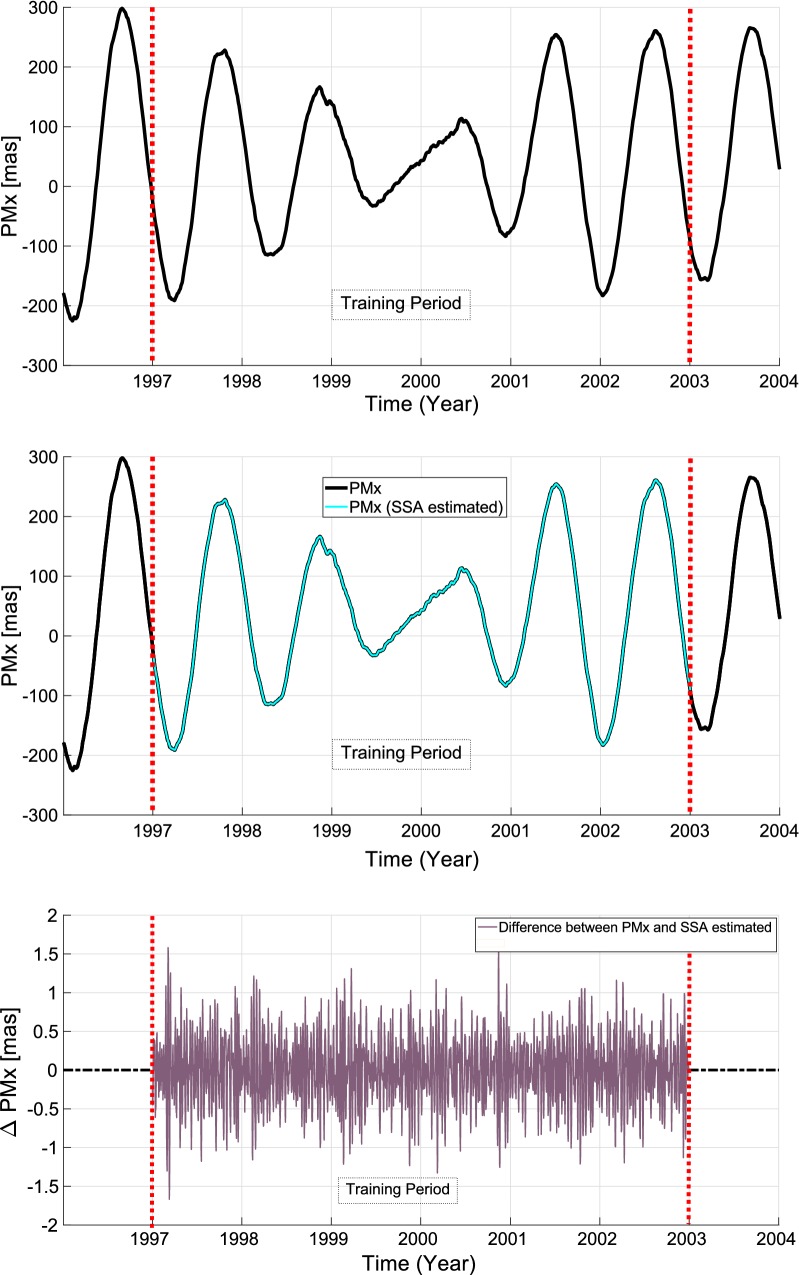
The original time series (upper panel), the reconstructed time series (middle panel), and the difference between original and reconstructed time series (lower panel) for ${\hbox{PM}}_{x}$

**Fig. 9:**
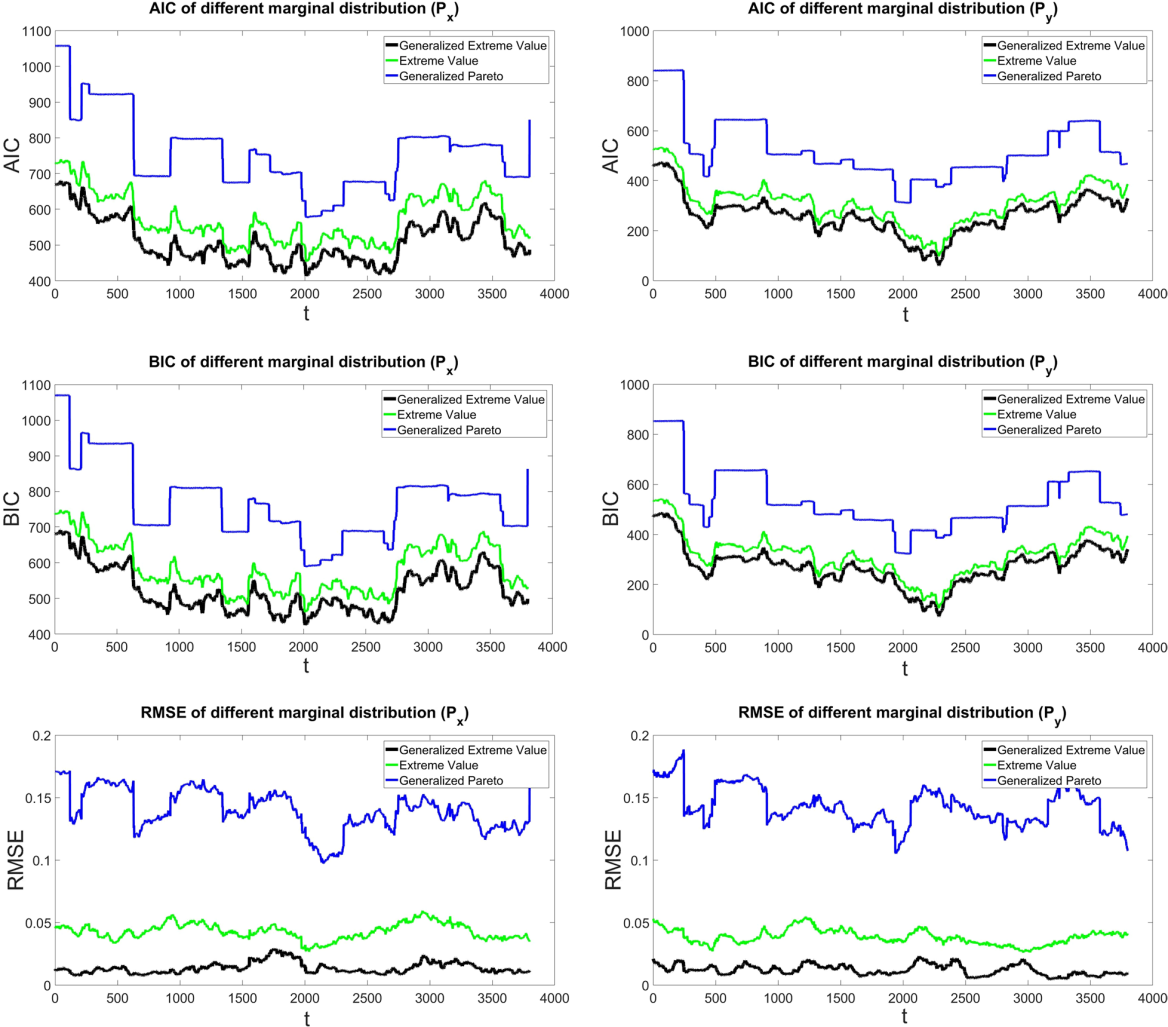
Marginal distribution’s goodness-of-fit test for ${\hbox{PM}}_{x}$ (left) and ${\hbox{PM}}_{y}$ (right). Generalized extreme value distribution is the black curve, green shows the extreme value distribution, and the blue curve is generalized Pareto distribution

**Fig. 10:**
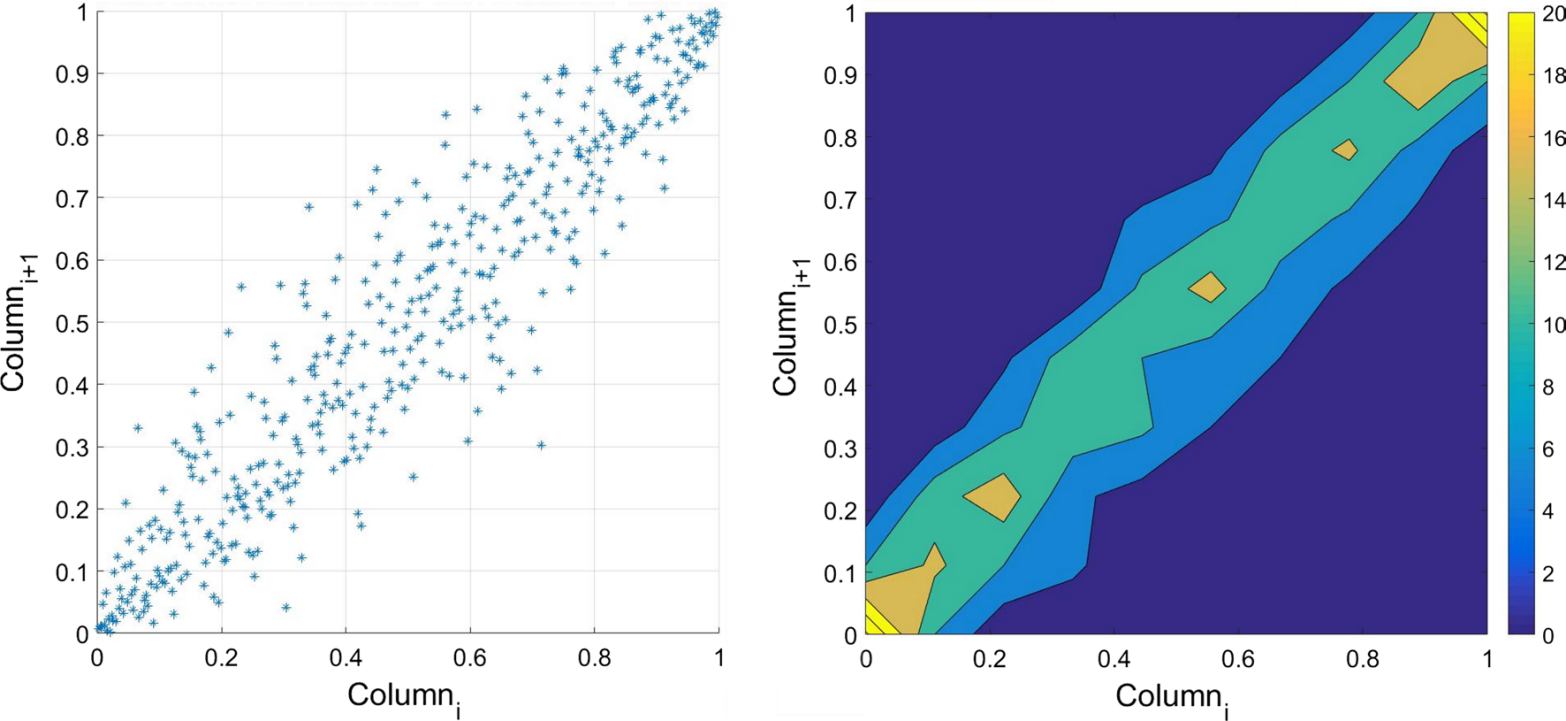
Scatter plot (left) two adjacent columns in the residual matrix. The empirical Copula (right) is estimated based on the dependency structure of two columns

PM is the sum of two statistically independent parts: trend and undulation. This hybrid model consists of a deterministic annual and the Chandler component as well as long-term lower-frequency parts which are estimated by SSA. The difference between the deterministic solution and the PM data is then used in a Copula-based model to predict stochastic processes. Then, the final PM prediction is a combination of the deterministic prediction (derived from the SSA solution) and the stochastic prediction (obtained from the Copula solution). To this end, first, the time series of PM from EOP 08 C04 were analyzed, and the trend is modeled and separated by SSA. Then, a Copula prediction model is made based on the SSA-separated time series. Finally, the accuracy of the proposed combined method is verified through different sets of PM prediction tests.

**Fig. 11:**
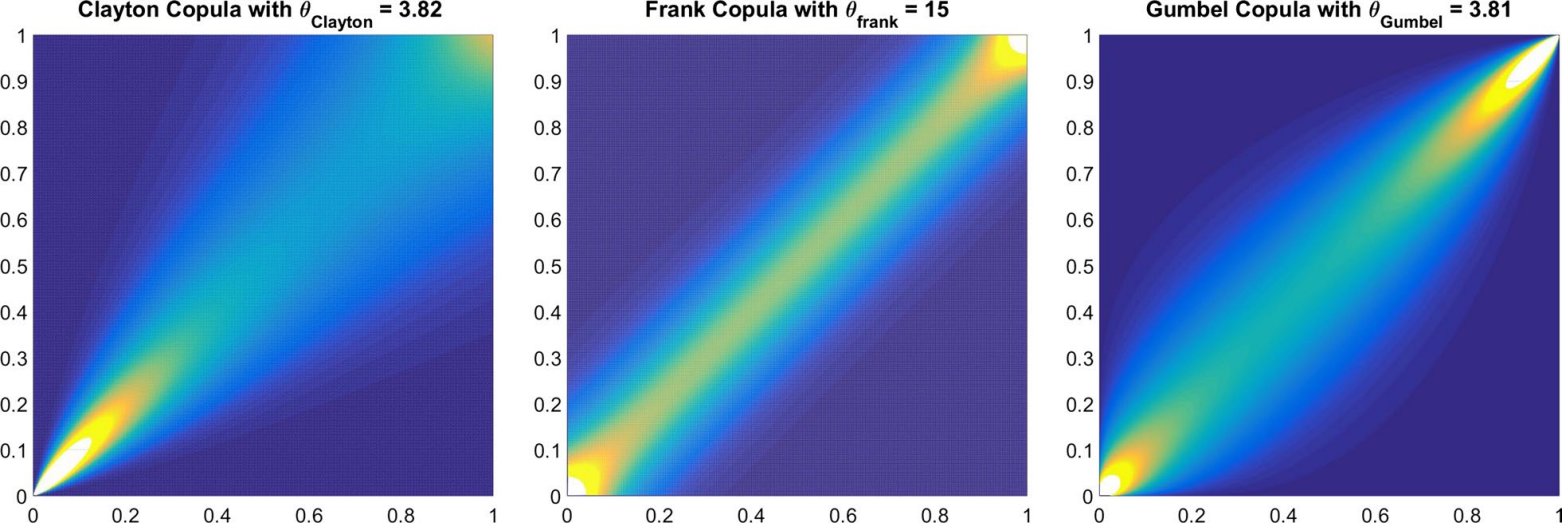
Theoretical Copula is fitted to the empirical Copula. The Copula parameter is 3.82, 15, and 3.61 for the Clayton, Frank, and Gumbel Copula, respectively

**Fig. 12:**
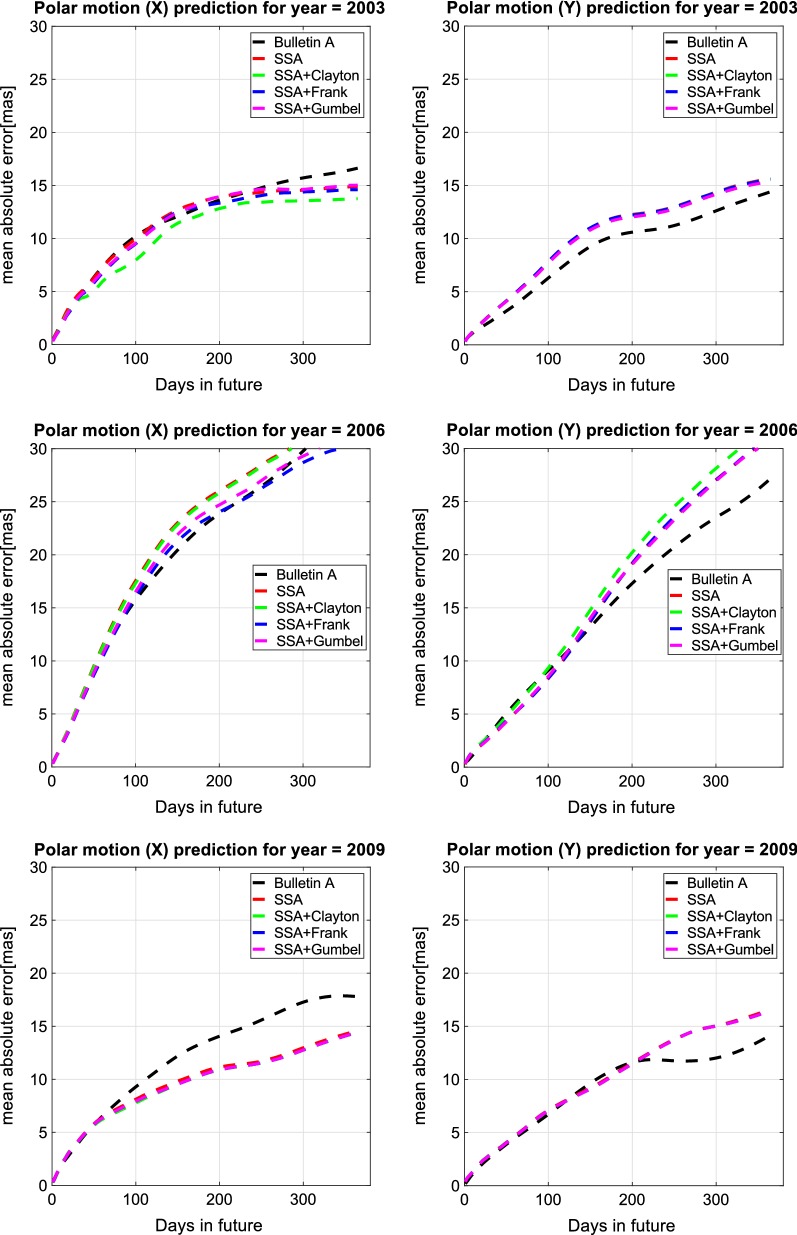
Mean value of MAE of ${\hbox{PM}}_{x}$ and ${\hbox{PM}}_{y}$ prediction for 2003, 2006, and 2009 with the unit [mas]

**Fig. 13:**
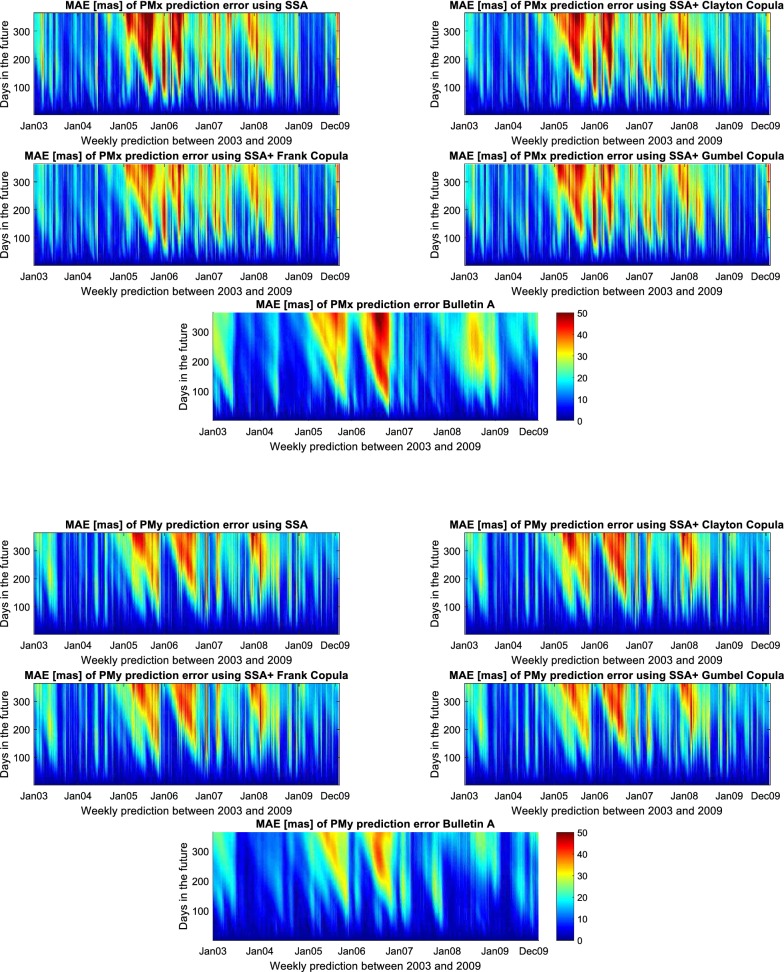
Absolute errors of the predicted ${\hbox{PM}}_{x}$ (up) and ${\hbox{PM}}_{y}$ (down) using SSA, SSA+Gumbel Copula, SSA+Clayton Copula, SSA+Frank Copula compared with Bulletin A product. The unit is [mas]

**Fig. 14:**
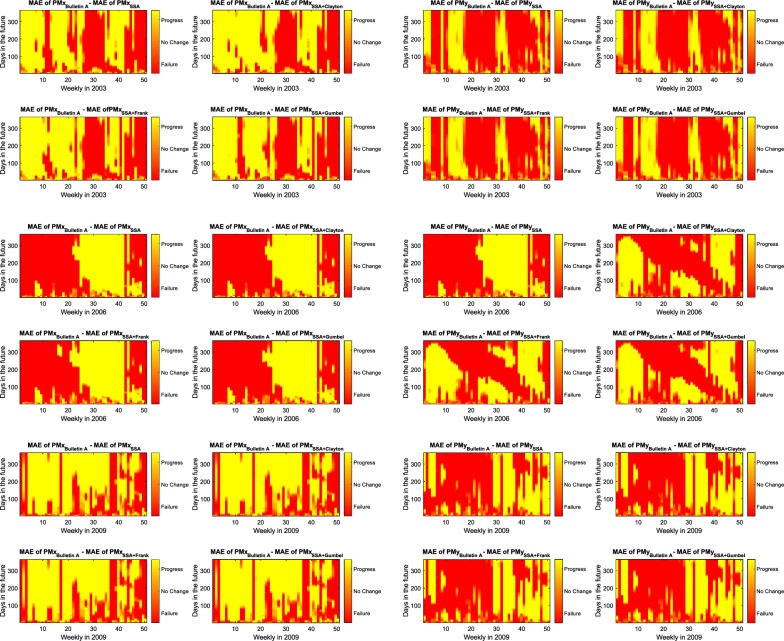
Improvement of ${\hbox{PM}}_{x}$ and ${\hbox{PM}}_{y}$ prediction using SSA + Copula-based model compared with Bulletin A product for 2003, 2006, and 2009. The improvement in prediction is shown by yellow color

## Methodology

In this study, we developed and explored the integration of Copula-based analysis and SSA for precisely predicting PM.

### Singular spectrum analysis

To maximize the prediction performance, we need a mathematical tool to retrieve all time-correlated information from the time series. As a matter of fact, the existence of excitations of PM can profoundly affect the forecasting procedure, particularly in longer intervals. Therefore, the exploitation of efficient techniques is crucial to minimize the risk of having gross errors.

SSA is a nonparametric spectral estimation method which can be used for decomposing a time series into the sum of interpretable components, e.g., trend, periodic components, and noise, without a priori assumption about the constituent components (Golyandina et al. 2001).

SSA is able to remove redundancies and groups uncorrelated information into informative empirical functions which can reveal main aspects of the time series. The mentioned functions are used as bases of a subspace in which the time series is a member of and can be exploited for modeling the time series in a desired level of details. Therefore, the model can simulate the future entries of the time series using these base functions.

The SSA method for trend extraction can be succinctly expressed as two stages:

#### *Decomposition*

First the time series is embedded in an L-dimensional vector space. The outcome of this stage will be a trajectory matrix ($\mathbf X$) which consists of L rows. The matrix has been simply formed using L-element lagged vectors taken from the time series by sliding a window of size L.

1 $$\begin{aligned}&\overbrace{x_1,x_2,\ldots,x_{L}}^{{\hbox {window}}\;\rightarrow },x_{L+1},\ldots ,x_N \; \Rightarrow \; X_1^T=(x_1,x_2,\ldots ,x_{L}) \end{aligned}$$

2 $$\begin{aligned}&x_1,\overbrace{x_2,\ldots ,x_{L},x_{L+1}}^{{\hbox {window}}\;\rightarrow },\ldots ,x_N \Rightarrow X_2^T=(x_2,x_3,\ldots ,x_{L+1}) \end{aligned}$$

3 $$\begin{aligned}&{{\mathbf {X}}}=[X_{1} \; X_{2} \; X_{3} \; \ldots \; X_{K}]={\begin{bmatrix}x_{1}&x_{2}&x_{3}&\ldots&x_{{K}}\\x_{2}&x_{3}&x_{4}&\ldots&x_{{K+1}}\\x_{3}&x_{4}&x_{5}&\ldots&x_{{K+2}}\\\vdots&\vdots&\vdots&\ddots&\vdots \\x_{{L}}&x_{{L+1}}&x_{{L+2}}&\ldots&x_{{N}}\\\end{bmatrix}} \end{aligned}$$

being $$1< L < K$$ and $$K = N-L+1$$.

Having the trajectory matrix formed, singular value decomposition (SVD) is applied to factorize $\mathbf{X}$ in the form of ${\mathbf {U\Sigma V^T}}$ in order to retrieve its principal components (PC).

4 $$\begin{aligned} {\mathbf {X}} = {\mathbf {U\Sigma V^T}} \end{aligned}$$

where ${\mathbf {U}}$ and ${\mathbf {V}}$ are the left and right singular vectors, respectively, and ${\varvec{\Sigma }}$ is a diagonal matrix consisting of singular values of $\mathbf{X}$ which reflect the importance of each corresponding pair of left–right singular vectors. The decomposition step can be performed using calculation of *eigenvalues* and *eigenvectors* of the matrix ${\mathbf {S}} = {\mathbf {XX^T}}$.

$$\begin{aligned} {\mathbf {XX^T}} = {\mathbf {(U \Sigma V^T)(U\Sigma V^T)^T = U \Sigma ^2 U^T = U \Lambda U^T}} \end{aligned}$$

Let $\lambda _1 \ge \lambda _2 \ge \cdots \ge \lambda _L \ge 0$ denote diagonal entries of ${{\varvec{\Lambda }}}$ (the eigenvalues of $\mathbf{S}$) and $U_1,U_2,\ldots ,U_L$ indicate the corresponding eigenvectors of $\mathbf{S}$ which are also called empirical orthogonal functions (EOF) of $\mathbf{X}$. The right singular vectors of $\mathbf{X}$ are eigenvectors of ${\mathbf {X^TX}}$ calculated by:

5 $$\begin{aligned} V_i = \mathbf{X}^T U_i/\root \of {\lambda _i}, \; \left\{ \begin{array}{l} d = \max \{ { i \; | \; \lambda _i>0} \} \\ i=1,2,\ldots ,d \end{array} \right. \end{aligned}$$

Now, the trajectory matrix can be written as:

6 $$\begin{aligned} {\mathbf {X}}={\mathbf {X_1+X_2+\cdots +X_d, \; X_i}}= \root \of {\lambda _i} U_i V_i^T \end{aligned}$$

#### *Reconstruction*

This stage aims to rebuild the time series using the reconstructed version of trajectory matrix. So, a subset of ${\mathbf {A}}=\{{\mathbf {X_1, X_2, \ldots , X_d}}\}$ can be chosen for reconstruction of the trajectory matrix. The choice of PCs of $\mathbf{X}$ defines how smooth would be the reconstructed version of the time series and how much detail of the original time series would be captured. Having a proper selection of PCs, a representative trend is extracted by applying diagonal averaging to the reconstructed trajectory matrix ($\hat{\mathbf{X}})$. Let $L < K$, and then, the trend of the time series $G = (g_1,g_2,\ldots ,g_N)$ is:

7 $$\begin{aligned} g_i = \; \left\{ \begin{array}{l} \frac{1}{i} \displaystyle {\sum \limits _{m=1}^{i}} {\hat{x}}_{m,i-m+1} \qquad \qquad \quad \; \; 1 \le i< L \\ \frac{1}{L} \displaystyle {\sum \limits _{m=1}^{L}} {\hat{x}}_{m,i-m+1} \qquad \qquad \quad \; L \le i \le K \\ \frac{1}{N-i+1} \displaystyle {\sum \limits _{m=i-K+1}^{N-K+1}} {\hat{x}}_{m,i-m+1} \qquad K < i \le N \end{array} \right. \end{aligned}$$

### Copula-based analysis

There is a well-introduced method called Copula that can be applied for polar motion modeling, estimation, and prediction. The word of Copula is a Latin noun that means a link or tie. The Copula method exploits linear and nonlinear dependency between variables. It is a potent and efficient tool for dealing with multi-dimensional data and modeling the relation between parameters based on the marginal distribution functions of the variables (Embrechts et al. 2002). Copula appeared in the context for the first time by Sklar (1959). Sklar’s theorem indicates that a Copula function *C* connects a given multivariate distribution function with its univariate marginal. For bivariate distribution, there is a bivariate Copula *C* which models the joint cumulative probability distribution function of two variables *X* and *Y* based on the marginal cumulative distribution functions $F_{X} (x)$ and $F_{Y}(y)$.

8 $$\begin{aligned} P(X\le x, Y\le y)&= F_{X,Y}(x, y) =C(F_X(x), F_Y(y))\nonumber \\ &= C(u, v) \end{aligned}$$

where *C* describes the joint distribution function $F_{X,Y}(x, y)$.*u* and *v* are transformed of *X* and *Y* to uniform distribution, respectively. Then, Joe (1997) and Nelsen (2007) developed the idea of the Copula. For many years, the Copula method has been used for modeling the dependence structure between random variables in different types of studies, e.g., economics (Rachev and Mittnik 2000; Patton 2006, 2009), biomedicine (Wang and Wells 2000; Escarela and Carriere 2003), hydrology (Bárdossy and Li 2008; Bárdossy and Pegram 2009; Verhoest et al. 2015), meteorology (Laux et al. 2011; Vogl et al. 2012), hydro-geodesy (Modiri et al. 2015). A brief introduction to the concept of copula function is given in the next subsections.

#### Characteristic of Copulas

In the bivariate case, a Copula is represented as a function *C* from $[0, 1]^{2}$ to [0, 1] so that $\forall u, v \in [0, 1]$ (Genest and Rivest 1993; Jaworski et al. 2010):

9 $$\begin{aligned} C(u, 0)&= {} C(0, v) = 0, \end{aligned}$$

10 $$\begin{aligned} C \left( u, 1 \right)&= {} u \quad {\text {and}} \quad C \left( 1, v \right) = v. \end{aligned}$$

Copula is an increasing function. It implies that $\forall u_1, u_2, v_1, v_2 \in [0,1] \ \ {\text {with}} \ \ u_1 \le u_2 \ \ {\text {and}} \ \ v_1 \le v_2$ holds

11 $$\begin{aligned} C(u_2, v_2)- C(u_2, v_1)- C(u_1, v_2)- C(u_1, v_1) \ge 0 \end{aligned}$$

Copula is a continuous function:

12 $$\begin{aligned} \vert C(u_2, v_2)- C(u_1, v_1)\vert \le \vert u_2-u_1\vert + \vert v_2-v_1\vert \end{aligned}$$

The Copula density is computed by differentiating Copula cumulative distribution function.

13 $$\begin{aligned} c(u, v) = \frac{\partial ^2 C(u,v)}{\partial u \partial v} \end{aligned}$$

#### Empirical Copula

The empirical Copula is an estimator for the unknown theoretical Copula distribution, and it is defined in the rank space as follows (Genest and Rivest 1993; Genest and Favre 2007; Laux et al. 2011):

14 $$\begin{aligned} C_e(u,v)=\frac{1}{n} \sum _{i=1}^n \mathbf{1 } \left( \frac{r_i}{n+1} \le u,\frac{s_i}{n+1} \le v\right) \end{aligned}$$

where,

- $(r_1), (r_2) \ldots , (r_n)$ denote the pairs of ranks of the variable $(x_1),(x_2), \ldots , (x_n)$,
- $(s_1), (s_2) \ldots , (s_n)$ denote the pairs of ranks of the variable $(y_1),(y_2), \ldots , (y_n)$,
- *n* is the length of the data vector,
- **1**(...) is the indicator function. If the condition is true, the indicator function is equal to 1. Otherwise, the indicator function is equal to 0.

### Archimedean Copula

A number of Copulas can be estimated directly with the simple form. They are named Archimedean Copulas. An Archimedean Copula can be presented in the following form:

15 $$\begin{aligned} C(u, v)= \phi ^{-1}\lbrace \phi (u)+\phi (v),\theta \rbrace \end{aligned}$$

where $\theta$ is the Copula parameter and the function $\phi$ is the generator of the Copula with the following characteristics (Nelsen 2007):

- for all $u \in (0,1), \phi (u) < 0$, $\phi$ is decreasing,
- for all $u \in (0,1), \phi (u) < 0$, $\phi$ is convex,
- $$\phi (1)=0$$,

and $\phi ^{-1}$ is defined by

$$\begin{aligned} \phi ^{-1}(t)= {\left\{ \begin{array}{ll} \phi ^{-1}(t;\theta ),&\quad {\text {if}} \quad 0\le t \le \phi (0)\\ 0, &\quad {\text {if}} \quad \phi (0)\le t \le \infty \end{array}\right. } \end{aligned}$$

There are three commonly used Archimedean Copula which are explained as follows and will be investigated in this study (see Table 1).

1. *Clayton Copulas*
  The generator of the Clayton Copula (see Fig. 1) is given by
  16 $$\begin{aligned} \phi ^{Cl}(x)=\frac{1}{\theta }(t^{-\theta }-1) \end{aligned}$$
  Therefore, the cumulative distribution function (CDF) for Clayton Copula is defined as (Clayton 1978):
  17 $$\begin{aligned} C_\theta (u,v)= \max [(u^{-\theta }+v^{-\theta }-1),0]^{-\frac{1}{\theta }} \end{aligned}$$
  where $\theta$ is restricted on the interval $[-1,\infty )$. If $\theta = 0$, it shows the independence case and when $\theta \rightarrow \infty$, indicate high dependency in the lower rank space.
2. *Frank Copula*
  The generator of the Frank Copula (see Fig. 2) is given by
  18 $$\begin{aligned} \phi ^{Fr}(t)=- \ln \left\{ \frac{\mathrm{e}^{-\theta t}-1}{\mathrm{e}^{-\theta }-1}\right\} \end{aligned}$$
  The parameter $\theta$ is defined over $\in (-\infty , \infty )-\lbrace 0 \rbrace$. The CDF for Frank Copula is given by (Joe 1997; Lee and Long 2009)
  19 $$\begin{aligned} C_\theta (u,v)= \frac{1}{\theta }\ln \left( 1+ \frac{(\mathrm{e}^{-\theta u}-1)(\mathrm{e}^{-\theta v})}{\mathrm{e}^{-\theta }-1}\right) \end{aligned}$$
  Frank Copula allows to model data with positive and negative dependency. The large positive and negative $\theta$ indicate high dependency, and $\theta = 1$ implies total independence. The Frank Copula is a suitable method for two data sets with the same dynamic characteristics (Rodriguez 2007).
3. *Gumbel Copulas*
  Gumbel Copula (see Fig. 3) is famous for its ability to capture strong upper tail dependence and weak lower tail dependence. Gumbel Copula is used to model asymmetric relationship in the data (Jaworski et al. 2010). The Gumbel Copula generator is written as:
  20 $$\begin{aligned} \phi (t)^{\rm Gu}=(-\ln t)^\theta \end{aligned}$$
  The CDF for Gumbel Copula is given by (Nelsen 2007)
  21 $$\begin{aligned} C_\theta (u,v)= \mathrm{e}^{-((-\ln (u)^\theta )+(-\ln (v)^\theta ))^{\frac{1}{\theta }}} \end{aligned}$$
  The Copula parameter $\theta$ is on the interval $[1, +\infty )$. If $\theta$ is equal 1, Copula shows independence. When $\theta \rightarrow \infty$, the Gumbel Copula indicates high dependence between the random variables.

**Table 1:** Three ordinary families of Archimedean Copulas (Clayton, Frank, and Gumbel Copula) and their generator, parameter space, and their formula

Family	Generator	Parameter	Formula
Clayton	$\phi ^{Cl}(x)=\frac{1}{\theta }(t^{-\theta }-1)$	$-\,1 \le \theta$	$C_\theta (u,v)= \max [(u^{-\theta }+v^{-\theta }-1),0]^{-\frac{1}{\theta }}$
Frank	$\phi ^{Fr}(t)=-\ln \left\{ \frac{\mathrm{e}^{-\theta t}-1}{\mathrm{e}^{-\theta }-1}\right\}$	$-\,\infty< \theta <\infty$	$C_\theta (u,v)= \frac{1}{\theta }\ln (1+ \frac{(\mathrm{e}^{-\theta u}-1)(\mathrm{e}^{-\theta v})}{\mathrm{e}^{-\theta }-1})$
Gumbel	$\phi (t)=(-\ln t)^\theta$	$1 \le \theta$	$C_\theta (u,v)= \mathrm{e}^{-((-\ln (u)^\theta )+(-\ln (v)^\theta ))^{\frac{1}{\theta }}}$

$\theta$ is the parameter of the Copula called the dependence parameter, which measures the dependence between the marginal

#### Copula parameter estimation

The widely used estimation method for the Copula parameter is the maximum likelihood (ML) estimation methodology (Joe 1997). The Copula parameters in this study are derived from ML estimation. The canonical maximum likelihood estimation (CLME) and inference for margins estimation (IFME) are two methods for estimation of the Copula parameter (Joe and Xu 1996). For both methods, the first step is marginal distribution estimation. Then, a pseudo-sample of the transformed observation is used to estimate the Copula parameter. In the IFME method, the theoretical marginal distribution parameters are estimated, and in the CMLE the univariate marginals are the empirical distribution functions (Giacomini et al. 2009). It is assumed that the sample data $(X_1, X_2, X_3, \ldots , X_n)$ are n independent and identically distributed (iid) random variables. These data are transformed into uniform variates $(r_1, r_2, r_3, \ldots , r_n)$.

Let $c(r_1, r_2, r_3, \ldots , r_n)$ be the density function of Copula $C(r_1, r_2, r_3, \ldots , r_n;\theta )$, and let $\theta$ be the Copula parameter which is estimated by maximizing the ML equation:

22 $$\begin{aligned} {\hat{\theta }} = \arg \max _{\theta }\sum _{i=1}^n \log c(r_1, r_2, r_3, \ldots , r_n; \theta ) \end{aligned}$$

#### Computation of conditional CDF for Archimedean Copula

In this subsection, the conditional CDF of Clayton, Frank, and Gumbel Copula are computed (Yue 1999; Zhang and Singh 2007; Trivedi et al. 2007). The conditional CDF for Clayton Copula is given by (Joe 1997):

23 $$C_{V=v}^{{\mathrm{Clayton}}}(u,v) = u^ {-\theta - 1}(-1 + u^{-\theta } + v^{-\theta })^ {(\frac{-1}{\theta } - 1)}$$

and for Frank Copula:

24 $$C_{V=v}^{{\mathrm{Frank}}}(u,v) = \frac{{\mathrm{e}}^{-u\theta }(-1+{\mathrm{e}}^{-v \theta })}{(-1+{\mathrm{e}}^{-\theta })\left( 1+\frac{(-1+{\mathrm{e}}^{-u \theta })(-1+{\mathrm{e}}^{-v\theta })}{-1+e{-\theta }}\right) }$$

The conditional CDF of Gumbel Copula is:

25 $$C_{V=v}^{\mathrm{Gumbel}}(u,v) = \frac{(-\ln u ^{\theta - 1})\left( \ln v^{(\theta - \ln v ^{\theta })^{\left( \frac{1}{\theta }-1\right) }}\right) }{{u e(- \ln u^{(\theta + \ln v^{\theta })^{\left( \frac{1}{\theta }\right) }}})}$$

#### Simulating from Copula-based conditional random data

This subsection provides the essential steps for data simulation using Copula-based conditional random data. The following steps are taken to fit the suitable theoretical Copula function and simulation data (Laux et al. 2011; Vogl et al. 2012).

1. Independent identical distribution (iid)-transformation of input time series.
2. Compute the marginal distribution $F_X (x)$ and $F_Y (y)$ of the input data *x* and *y*.
3. Transform data to rank space using the estimated marginal distributions of data with $u_i$ and $v_i$ in rank space.
4. Compute the empirical Copula to the dependence structure of random variables using the rank-transformed data.
5. Fit a theoretical Copula function $C_\theta (u,v)$.
6. Compute the conditional Copula function.
7. Sample random data from the conditional Copula CDF.
8. Transfer the sample back to the data space using the inverse marginal.

### Error analysis

The mean absolute error (MAE) standard is used in order to evaluate the prediction accuracy. It can be shown as follows:

26 $$E_{i}= P_{i}-O_{i}$$

27 $$\mathrm{MAE}= \frac{1}{n}\sum _{i=1}^n(|E_{i}|)$$

where $P_{i}$ is the predicted value of the *i*-th prediction, $O_{i}$ is the corresponding observation value, $E_{i}$ is the error, and *n* is the total prediction number (Willmott and Matsuura 2005).

## Calculation and analysis

### Data description

In this paper, the ${\hbox{PM}}_{x}$ and ${\hbox{PM}}_{y}$ time series (see Fig. 4) are from the International Earth Rotation and Reference Systems Service (IERS) combined earth orientation parameter (EOP) solutions 08 C04 (available at http://hpiers.obspm.fr/eop-pc/analysis/excitactive.html). The EOP 08 C04 series is derived from different geodetic techniques, and it is consistent with ITRF 2008. The EOP 08 C04 time series cover the period 1962 to the present. The sampling interval is one day.

### Data processing and analysis

In this study, we defined an algorithm for PM prediction which is shown in Fig. 5. The observed PM time series can be split up into two parts. The first part is dealing with periodic effects such as Chandler wobble, annual variation, and influences of solid Earth tides and ocean tides on PM. The SSA is used to model the periodic terms of the PM. Then, the difference between the observed PM and SSA estimated data is modeled by using the Copula-based analysis method. After that, the periodic terms of PM are extrapolated using the SSA a priori model. Also, the anomaly part is predicted using the Copula-based model. Finally, the anomaly solution is added to the SSA-forecasted time series.

Therefore, the analysis of the data is divided into two main steps:

1. SSA Periodic Terms Estimation
  Selecting window parameter (L) considering the dominant periods of the time series and the prediction interval,
  Forming trajectory matrix ($\mathbf{X}$) using L,
  Singular value decomposition of $\mathbf{X}$,
  Selecting a proper group of singular values and corresponding singular vectors,
  Reconstruction of $$\mathbf{X}$$,
  Calculation of the trend by applying diagonal averaging to $\mathbf{X}$.
2. Copula Anomaly Modeling
  Subtract the observed PM time series by SSA-reconstructed time series,
  Forming the trajectory matrix of residual time series using window length L and time delay of 1 day,
  Compute the marginal distribution of each column of the matrix,
  Transform data to the rank space,
  Compute the empirical Copula between the column i and i+1,
  Fit the theoretical Copula model by applying appropriate goodness-of-fit tests,
  Compute the conditional Copula,
  Sample random data from the conditional Copula CDF and transfer the sample back to the data space using the inverse marginal,
  For each value of one input time series, one obtains an ensemble of possible values for other time series.

Therefore, the final PM predicted data is the sum of the results of predicted periodic terms using SSA and predicted anomaly using the Copula-based model.

### SSA periodic terms estimation

Window length selection is a crucial step in SSA which has a significant impact on the decomposition of the time series. The appropriate choice for *L* in a periodic time series with a period *T* is a window length proportional to the period, meaning that the *L* / *T* is an integer. Figure 6 depicts the main periods of PM time series (Golyandina and Zhigljavsky 2013). So, the Chandler period as the main period of both time series would be a reasonable choice. Making the closest choice to the half of the length of the time series (if possible, least common multiple of the Chandler and annual periods) is recommended by Golyandina and Zhigljavsky (2013), but is avoided due to the processing time.

After selection of the window length, the number of singular vectors or empirical functions for reconstruction of the time series should be determined. The goal of this procedure is to find and apply a proper set of constructive components. Most significant periodicities as well as excitation mechanisms are rather low-frequency components and reveal their impact in the first few singular vectors while high-frequency components fall in later singular vectors. The singular value spectrum reflects the importance of each singular vector. Figure 7 suggests that in order to achieve an accuracy of about 1 mas in polar motion modeling, we need to utilize at least first 70 singular vectors which correspond to using all components with periods more than or equal to 14 days.

Having the window length and the number of singular values determined, we construct the trajectory matrix. As it can be seen in Fig. 8 the data between the year of 1997 and 2003 is used as the training period. The cyan curve is the SSA-reconstructed ${\hbox{PM}}_{x}$ time series. Prediction of the future entries starts by adding initial guess of future entries to the end of the time series. Then, iteration of the SSA process is done until the result of two successive iterations has a difference less than a certain threshold. This will map the initial values to the estimated periodic terms of the time series. The residual part of the difference between original ${\hbox {PM}}_{x}$ time series and SSA estimated time series is named anomaly of ${\hbox {PM}}_{x}$ which has a stochastic behavior. Therefore, the anomaly part will be investigated by Copula-based technique.

### Copula anomaly modeling

The anomaly part which is shown in Fig. 8 (lower panel) with dark violet is formed into a matrix with the same window length L. Then, the dependency structure between the ${rm column}_{i}$ and ${rm column}_{i+1}$ is investigated for the whole dataset. Modeling the joint dependence structure with Copulas requires fitting marginal distribution to data. In this study, three univariate distribution functions are considered: extreme value, generalized extreme value, and generalized Pareto distribution (see Table 2). To identify which univariate distribution is the best suitable for both ${\hbox {PM}}_{x}$ and ${\hbox {PM}}_{y}$, the root-mean-square error is estimated and the goodness of fit is examined with the Akaike and the Bayesian information criteria (AIC and BIC).

28 $$\begin{aligned} \mathrm{AIC} = 2k-\ln (B) \end{aligned}$$

and

29 $$\begin{aligned} \mathrm{BIC} = k\ln (n)-2\ln (B) \end{aligned}$$

where *k* denotes the number of the free parameters in the model. *n* is the sample size, and *B* is the maximized value of the likelihood function of the estimated model. The smallest amount of AIC or BIC, respectively, suggests the best fitting model or distribution. After estimation of the parameters by maximum likelihood approach, the AIC, BIC, and RMSE values are calculated for both ${\hbox {PM}}_{x}$ and ${\hbox {PM}}_{y}$ distribution. As it can be seen in Fig. 9, the generalized extreme value (black) provides the best fit in comparison with the generalized Pareto distribution function (blue) and extreme value distribution function (green). Furthermore, according to Tables  3 and 4, the result of the AIC, BIC, and RMSE confirmed that the generalized extreme value provides the best fit in both ${\hbox {PM}}_{x}$ and ${\hbox {PM}}_{y}$ distribution. Therefore, generalized extreme value distribution was selected in this study.

**Table 2:** Marginal distributions

Distribution	Formula	Parameters
Extreme value (Kotz and Nadarajah 2000)	$f(x;\mu , \sigma )= \sigma ^{-1}\exp (\frac{x-\mu }{\sigma })\exp \left( -\exp \left( \frac{x-\mu }{\sigma }\right) \right)$	Location $\mu$ scale $\sigma$
Generalized extreme value (Hosking et al. 1985)	$${\displaystyle f(x;\mu , \sigma ,\xi )={{\left\{ \begin{array}{ll}{\big (}1+\xi ({\tfrac{x-\mu }{\sigma }}){\big )}^{-1/\xi }&{}{\text {if}}\ \xi \ne 0\\ \mathrm{e}^{-(x-\mu )/\sigma }&{}{\text {if}}\ \xi =0\end{array}\right. }}}$$	Location $\mu$ scale $\sigma$ shape $\xi$
Generalized Pareto (Hosking and Wallis 1987)	$f(x;\sigma ,\xi )= f_{{(\xi ,\mu ,\sigma )}}(x)={\frac{1}{\sigma }}\left( 1+{\frac{\xi (x-\mu )}{\sigma }}\right) ^{{\left( -{\frac{1}{\xi }}-1\right) }}$	Location $\mu$ scale $\sigma$ shape $\xi$

**Table 3:** Goodness-of-fit test for marginal distribution of ${\hbox {PM}}_{x}$

Distributions	AIC	BIC	RMSE
Extreme value	574.60	582.66	0.04
Generalized extreme value	511.14	523.23	0.01
Generalized Pareto	758.22	770.31	0.13

**Table 4:** Goodness-of-fit test for marginal distribution of ${\hbox {PM}}_{y}$

Distributions	AIC	BIC	RMSE
Extreme value	310.99	319.05	0.03
Generalized extreme value	261.25	273.34	0.01
Generalized Pareto	523.99	536.09	0.14

### Estimating empirical Copula

Once the univariate marginal distribution is fitted, the dependence structure between the time series has to be investigated. The first step is to calculate the empirical Copula using Eq. (14). As it can be seen in Fig. 10, there is a scatter plot of two adjacent columns, and it shows a scatter linear dependency structure with the heavy tail. This kind of dependency structure can be correctly modeled using the Archimedean Copula.

### Fitting a theoretical Copula function

The next step is fitting a theoretical bivariate Archimedean Copula function with its parameters estimated by maximum likelihood approach. In this study, three different theoretical Copula functions are tested (Fig. 11): Clayton, Frank, and Gumbel Copula. For the three different Copula functions, the goodness-of-fit test, which is based on the Cramer–von Mises statistics, is applied. To evaluate the performance of the Copulas, 1000 values of the test statistics are sampled, and the proportion of values larger than $S_{n}$ is estimated by calculating the corresponding *p* values. The results based on $S_{n}$ show that the performance of Frank Copula is slightly better than Gumbel and Clayton Copula with less error (Table 5).

**Table 5:** Goodness-of-fit test for Copula model

Copula name	Clayton	Frank	Gumbel
Mean($S_{n}$)	43.57	12.13	17.58

### 365-day-ahead prediction

We utilized 6 years of observed PM time series, from January 1997 to December 2002, for the 365-day-ahead prediction. To verify the reliability of this method, the results were compared with the IERS Bulletin A predictions (https://datacenter.iers.org/web/guest/bulletins/-/somos/5Rgv/version/6). The IERS Bulletin A contains the PM parameters and the predicted PM for one year into the future, and they are released every seven days by IERS Rapid Service/Prediction Center (RS/PC), hosted by the U.S. Naval Observatory (USNO) (Petit and Luzum 2010; Gambis and Luzum 2011). The predictions of PM from the IERS Bulletin A were produced by LS + AR method. In the current prediction method, the PM prediction was the sum of the LS extrapolation model (including the Chandler period, annual, semiannual, terannual, and quarter annual terms), and the AR predictions of the LS extrapolation residuals (Kosek et al. 2007).

## Discussion of results

In this study, we demonstrated the PM prediction by combination of SSA and Copula-based analysis method. Our method is tested based on the hindcast experiments using data from the past. Hence, we have calculated the results of our methods yearly for seven years of the test period 2003–2009 in comparison with Bulletin A PM prediction. As the prediction solutions of Bulletin A are available weekly, we would have approximately 52 time series of prediction for each year. So, Fig. 12 shows the mean value of MAE for each year. In Fig. 12, the Bulletin A solution is shown in black and the SSA predicted data in red. Also, the results of SSA+Copula are displayed by green, blue, and pink for Clayton, Frank, and Gumbel Copula, respectively. Compared to the results from the IERS Bulletin A, the MAE of the predictions produced by the proposed method was smaller in different short-, mid-, and long-term intervals for different cases (e.g., between 1 and 5 mas progression of ${\hbox {PM}}_{x}$ prediction for different time intervals in 2003). The better prediction performance of the SSA + Copula prediction may have been due to the modeling of the linear change of the Chandler and the annual oscillation amplitudes. Besides, the combination of SSA+Copula improves the SSA solution because of its ability to model the stochastic behavior of the anomaly part of the PM time series. However, the proposed method did not always perform better, especially in cases of long-term prediction where the quality of the results was not as good as we expected (see Fig. 12). This may have been caused by changes of the amplitudes of the periodic terms in this six-year time span where the SSA was not able to capture all features in order to predict more precisely we would have to increase the interval of training time. Figure 13 presents the absolute error of 365-day-ahead prediction between 2003 and 2009. Different patterns and features can be seen in our solution and Bulletin A solution. For instance, Bulletin A predicted ${\hbox {PM}}_{x}$ from January to March 2003 displays errors of more than 30 mas which cannot be found in our results, and there is a clear feature in ${\hbox {PM}}_{x}$ Bulletin A mean absolute error plot from August to December 2008 which does not appear in our prediction. However, our predicted PM results indicate a periodic error in mid- and long-term predictions although the results of the combination SSA + Copula show smoother errors in comparison with the SSA results. To better understand this particular periodic error of our method, we plot Fig. 14 that demonstrates the improvement in the SSA + Copula predicted solution compared to Bulletin A. For each prediction epoch, if the difference between errors of Bulletin A prediction and errors of SSA/SSA+Copula is positive, it is considered as an improvement in prediction. Yellow color shows the progress in prediction in heat maps (see Fig. 14). The red color indicates where our method shows higher errors than Bulletin A in the prediction process. Also, the orange shows where both PM prediction techniques display the same amount of error. The results illustrate that SSA+Copula can improve the accuracy of PM prediction in the different time intervals of prediction (short, mid, and long). Tables 6 and 7 indicate the success rate of PM prediction when using the SSA + Copula algorithm. The success rate of PM prediction is illustrated by the number of improvement in PM prediction (yellow) over the total number of PM prediction (yellow+ orange+ red).

30 $$\begin{aligned} {\text {Success rate of PM prediction}} = \frac{\text {Number of improvement in the predicted PM }}{\text {Total number of PM prediction}}\times 100 \end{aligned}$$

The improvement in the prediction is approximately 40% on average. According to Malkin and Miller (2010), there is Candler Wobble phase variation in 1850, 1925, and 2005. So, probably it is the reason why the proposed prediction method losses accuracy around the year 2005. Also, as it can be seen in Tables 6 and 7 the success rate of ${\hbox {PM}}_{x}$ and ${\hbox {PM}}_{y}$ can be reached up to 64.99 and 46.66%, respectively.

**Table 6:** Success rate of ${\hbox {PM}}_{x}$ prediction [%]

Method$\backslash$year	2003	2004	2005	2006	2007	2008	2009	Average
SSA	55.29	33.31	26.52	40.16	22.90	45.94	64.70	41.26
SSA + Clayton	61.71	33.88	31.91	40.17	22.91	45.96	64.95	43.07
SSA + Frank	58.31	34.31	33.61	42.50	22.91	45.97	64.99	43.22
SSA + Gumbel	55.90	33.81	28.31	41.00	22.90	45.94	64.97	41.83

**Table 7:** Success rate of ${\hbox {PM}}_{y}$ prediction [%]

Method$\backslash$year	2003	2004	2005	2006	2007	2008	2009	Average
SSA	35.95	44.99	25.43	45.28	39.50	29.21	39.50	37.12
SSA + Clayton	35.99	44.84	24.93	41.27	39.57	29.14	39.65	36.48
SSA + Frank	35.94	44.82	25.54	46.66	39.45	29.30	39.70	37.36
SSA + Gumbel	38.45	44.60	26.66	44.46	39.36	29.55	39.74	37.54

## Conclusions

The improvement in the Earth rotation prediction is a relevant, timely problem, as confirmed by the fact that the International Astronomical Union (IAU) Commission A2, the International Association of Geodesy (IAG), and the IERS have at present two Joint Working Groups on Prediction (JWG-P) and on Theory of Earth rotation and validation (JWG-ThER). According to the United Nations (UN) resolution in 2015, the primary objective of these JWGs is to assess and ensure the level of consistency of earth orientation parameter (EOP) predictions derived from theories with the corresponding EOP determined from analyses of the observational data provided by the various geodetic techniques. Therefore, accurate EOP predictions are essential to avoid any systematic drifts and/or biases between the international celestial and terrestrial reference frames (ICRF and ITRF). The results illustrate that the proposed method could efficiently and precisely predict the PM parameters. As clearly demonstrated, the SSA + Copula algorithm shows better performance for ${\hbox {PM}}_{x}$ prediction in comparison with the SSA prediction. The Copula-based analysis is fully successful in its aim to increase the accuracy of PM prediction by modeling the stochastic part of the PM and subtracting PM by SSA-reconstructed time series. We suspect the main error contributions come from SSA extrapolation part. So, further investigations about the SSA training time will be required to clarify this issue. Also, SSA + Copula prediction method shows periodic errors, and these errors have a significant impact on the mean absolute error. Therefore, these occasional errors should be further investigated to have a noticeable progression in the PM prediction accuracy.

## Abbreviations

ANN: artificial neural network
AR: autoregressive
CDF: cumulative distribution function
CLME: canonical maximum likelihood estimation
DORIS: Doppler Orbitography and Radiopositioning Integrated by Satellite
EOP: Earth orientation parameters
EOP PCC: Earth orientation parameters prediction comparison campaign
GFZ: German Research Centre for Geosciences
GGOS: Global Geodetic Observing System
GNSS: Global Navigation Satellite Systems
IAG: International Association of Geodesy
ICRF: international celestial reference frame
IERS: International Earth Rotation and Reference Systems Service
IFME: Inference for Margins Estimation
LLR: Lunar Laser Ranging
LS: least squares
MAE: mean absolute error
RMS: root-mean-square
PM: polar motion
TRF: terrestrial reference frame
VLBI: Very-long-baseline interferometry
SLR: Satellite Laser Ranging
SSA: singular spectrum analysis
